# Editorial: Novel methods to advance diagnostic and treatment value of medical imaging for cardiovascular disease

**DOI:** 10.3389/fbioe.2022.987326

**Published:** 2022-08-31

**Authors:** Zahra Keshavarz-Motamed, Juan C. Del Alamo, Danny Bluestein, Elazer R. Edelman, Jolanda J. Wentzel

**Affiliations:** ^1^ Department of Mechanical Engineering, McMaster University, Hamilton, ON, Canada; ^2^ School of Biomedical Engineering, McMaster University, Hamilton, ON, Canada; ^3^ Department of Mechanical Engineering, University of Washington, Seattle, WA, United States; ^4^ Center for Cardiovascular Biology, University of Washington, Seattle, WA, United States; ^5^ Department of Biomedical Engineering, Stony Brook University, Stony Brook, NY, United States; ^6^ Institute for Medical Engineering and Science, Massachusetts Institute of Technology, Cambridge, MA, United States; ^7^ Cardiology Department, Biomedical Engineering, Biomechanics Laboratory, Erasmus MC, Rotterdam, Netherlands

**Keywords:** diagnostic methods, medical imaging, cardiovascular disease, monitoring, bioengineering

The use of medical imaging has substantially increased over the past decade, thanks to the technological advancements evident from the dramatic improvement in the sensitivity and spatial resolution of imaging modalities. Cardiovascular imaging has been at a crossroads regarding technological advances, with a shift in focus from single-modality diagnosis to an integrated multimodality approach that can provide comprehensive assessments of morphology, pathophysiology, and disease biology to stratify the patient risk and guide therapies. The wide inter-subject variability in cardiovascular anatomy and pathophysiology urges the design of personalized patient management, which can highly benefit from clinical imaging technologies. The remarkable advances in medical imaging have sparked the development of new image processing algorithms and image-based simulation tools. In addition to providing comprehensive diagnostic information, some tools can even predict intervention outcomes, thereby enabling personalized intervention planning. This Research Topic, *Novel Methods to Advance Diagnostic and Treatment Value of Medical Imaging for Cardiovascular Disease*, focuses on tools that augment the power of medical imaging to provide detailed quantification of cardiovascular disease. Here, we present a Research Topic of 21 research articles that provide the reader with information regarding recent advancements in medical imaging improving diagnosis, prediction, monitoring, and treatment of cardiovascular disease.

## Coronary disease

Accurate coronary blood flow quantification is crucial in the proper diagnosis and treatment planning in patients with coronary artery disease. Swine animal models are invaluable to study coronary flow and develop predictive tools for disease initiation and progression. Coronary hemodynamics play a fundamental role in these processes but the correspondence between human and swine coronary flow has not been previously demonstrated. De Nisco et al. confirmed the validity of swine-specific computational models to characterize coronary hemodynamics parameters and diseases such as atherosclerosis and their translation to human vascular disease. This validation was performed using a comparative computational fluid dynamics analysis (CFD) between swine-specific and human-specific models. The analysis involved several flow and anatomical features obtained through intravascular ultrasound and coronary computed tomography angiography.

A study by Hair et al. investigated the importance of using both patient-specific anatomic and flow information from magnetic resonance angiography in order to accurately assess the functional significance of coronary lesions. The purpose of the study was to investigate the effects of varying coronary flow reserve values on the calculation of fractional flow reserve. State-of-the-art CFD analyses of coronary flow are performed on high-resolution patient-specific coronary anatomies from CT but use flow rates obtained from population statistics and allosteric scaling as inlet boundary conditions. The authors demonstrated that this approach provides fractional flow reserve values that differ from those calculated by CFD with patient-specific flow rates derived from magnetic resonance angiography data, demonstrating the importance of patient-specific features on coronary disease evaluation.

Several studies have been performed which build upon current diagnostic imaging modalities. Blanken et al. developed a framework which quantifies coronary flow using accelerated 4D flow MRI with respiratory motion correction compressed sensing image reconstruction. The developed framework improves upon the current use of 2D flow MRI which is limited in clinical applicability. The proposed framework allows for diastolic quantification of left coronary flow which agrees with 2D flow MRI. Current intravascular ultrasound (IVUS) parameters cannot accurately diagnose intermediate coronary stenosis. By integrating IVUS parameters with lesion length, Li et al. demonstrated the potential for accurate diagnosis of intermediate coronary stenosis using IVUS parameters.

Dang et al. investigated the correlation between the quality of pericoronary adipose tissue (PCAT) derived from dual-layer spectral detector CT (SDCT) and the severity of coronary artery disease. PCAT is a known contributor to the development of atherosclerosis and the authors of this study suggest that the use of SDCT for PCAT may be an important feature to monitor in the development of coronary artery disease.

## Valvular disease

Due to the wide range of differences in cardiovascular anatomy and pathophysiology, treatment planning for patients requiring valve repair or replacement calls for patient-specific approaches. Baiocchi et al. explored the effects of different imaging modalities, namely Doppler echocardiography and computed tomography, on a previously developed framework which diagnoses and monitors complex ventricular, vascular, and valvular disease for patients who undergo transcatheter aortic valve replacement (TAVR). Both of the mentioned imaging modalities along with the developed framework were compared against cardiac catheterization for patients with complex valvular, vascular, and ventricular disease who undergo TAVR in both pre and post-intervention. Schmid et al. investigated whether global indices of ventricular function measured by cardiac MRI pre-TAVR predict post-implantation outcomes. Magnetic resonance was right performed pre-TAVR to quantify volumetric function and global longitudinal and circumferential strain of both ventricles. The results of this study demonstrated that right ventricular function may play a role in predicting intermediate-term mortality in TAVR patients.

Dabiri et al. developed a near real-time, machine learning framework to predict the outcomes of MitraClip (MC) intervention for mitral regurgitation. Currently, fluid structure interaction simulations are used to predict outcomes of MC interventions, however, the high computational cost of these simulations make them unpractical in clinical settings. The machine learning method proposed in this study leverages patient-specific image data including 3D echocardiographic images, which are augmented using tools like principal component analysis to ultimately obtain stress maps and mitral regurgitation through the valve, enabling to predict the outcome of MC interventions in near real time.

Tetralogy of Fallot is a congenital heart defect which involves ventricular septal defect, pulmonary valve stenosis, aorta overriding, and right ventricular hypertrophy. Currently, this congenital disease is often surgically treated with pulmonary valve replacement (PVR) which carries risk as an open-heart operation. Yu et al. adapted computational biomechanical models to study the impact of PVR with five band insertions. Using CMR images, 147 computational bi-ventricle models were constructed to simulate right ventricle cardiac functions and identify optimal band treatment options. The results from this study showed great potential of using active contraction bands to improve ventricular function in patients with Tetralogy of Fallot.

## Vascular disease

Vascular disease in general is often onset due to abnormal interactions between vessel morphology and hemodynamics. De Marinis and Obrist developed a data assimilation methodology which can be used to improve spatial and temporal resolution of voxel-based flow data as obtained from biomedical imaging modalities. Combined with a CFD solver, this framework can be enhance the resolution of fine-scale flow features of cardiovascular flows.

Salmasi et al. investigated the impacts of blood flow patterns on the material properties of ascending thoracic aortic aneurysms from vascular remodeling. This was achieved by using image-based computational modelling to determine wall shear stresses along with *ex-vivo* measurements of tissue-derived mechanical and microstructural properties using segmental analysis.

An additional study by Saitta et al. explored the interactions between aortic morphology and hemodynamics in the development of type B aortic dissection (TBAD). Four patients with varying type B aortic dissection morphologies underwent both CT and 4D flow MRI imaging to perform flow visualization and quantitative analysis in the true and false lumens of the dissected aorta. This study demonstrated the clinical feasibility of 4D flow MRI in TBAD patients and its importance in assessing the hemodynamic footprint of this condition.

Lekavich et al. studied the effects of aerobic training compared to resistance training on cardiac and peripheral arterial capacity on cardiopulmonary and peripheral vascular function. Several parameters including strain-based variables, brachial artery flow-mediated dilation, as well as peak VO_2_ and peak O_2_ pulse were analyzed in sedentary and obese adults. The results of this study can lead to optimal choice of exercise modality to achieve specific clinical endpoints.

A crucial portion of the vascular system lies within the cranium to supply the brain with adequate oxygen. Image-based CFD analysis provides precise predictions of cerebral flow when supplied with inflow-outflow boundary conditions. However, the redundancy of flow paths offered by the circle of Willis makes it difficult to establish accurate patient-specific boundary conditions from anatomical images alone. Schollenberger et al. used arterial spin labeling MRI to solve this challenge in order to simulate cerebral hemodynamics in a patient-specific manner for patients suffering from cerebrovascular stenoses.

## Myocardial/ventricular disease

Myocardial and ventricular disease have major implications on the entire cardiovascular system and must be treated promptly and properly in order to provide the best outcome for each patient. Huang et al. investigated alterations in left ventricular myocardial workload using the left ventricular pressure-strain loops in patients with type 2 diabetes mellitus. In this study, various biomechanical features including global longitudinal strain, global work index, global constructive work, and left ventricular ejection fraction were evaluated. Patient specific parameters were measured using echocardiography, 2-D speckle tracking echocardiography (STE), and LV pressure strain-loop (LVPSL) technology.

Myocardial infarction (MI) is caused by a lack of oxygen supplied to the myocardial cells and is a major cause of death and disability worldwide Kotronias et al. investigated the use of angiography-derived index of microcirculatory resistance to predict microvascular injury in patients with ST-segment-elevation MI. Current use of pressure-wire based methods to predict microvascular injury remain costly and procedurally complex. Peng et al. reviewed magnetic resonance texture analysis in MI, summarizing the outstanding challenges and clinical applications and illustrating how image-based metrics are increasingly used as biomarkers in the diagnosis and prognosis of cardiovascular diseases.

Medical imaging of myocardial fibrosis, which is associated with impaired contractility, increased stiffness, and electrophysiological alterations, is receiving increasing attention in recent years. A study performed by Sun et al. used both 2-D and 3-D STE to evaluate myocardial strain as well as determine which method is a more robust predictor of myocardial fibrosis in heart transplant recipients. This was accomplished by evaluating myocardial fibrosis by CMR extracellular volume fraction and its association with myocardial strain, measured using the methods originally mentioned. The results of this study demonstrated the potential of both 2-D and 3-D STE to monitor the development of fibrotic remodeling following heart transplant. An additional study performed by Cui et al. used the non-invasive LVPSL method to measure global myocardial work indices in patients with dilated cardiomyopathy. The results of the study demonstrated that the parameters measured by non-invasive LVPSL can be significant predictors of myocardial fibrosis in patients with dilated cardiomyopathy.

The idea of LV flow as a biomarker was explored using 4-D flow MRI by Kim et al., who studied patients with paroxysmal atrial fibrillation (PAF) and normal LV function imaged in sinus rhythm. The authors found LV blood particles take longer to transit the LV in PAF patients when compared to controls, suggesting that LV flow could be a sensitive biomarker that can detect subtle phenotypic differences.

Cardiomyopathy, as shown by Morita et al., can be heavily influenced based on genetic testing. Genetic testing carries socioeconomical and psychological burdens and it is therefore important to identify patients with cardiomyopathy who are more likely to have a positive genotype. The model developed in this study demonstrates superior accuracy to predict positive genotypes in patients with cardiomyopathy compared to standard methods. The developed framework involves a deep convolutional neural network algorithm to analyze echocardiographic images.

## Conclusion and future directions

In the past 2 decades, the use of medical imaging has drastically increased, and medical imaging had astonishing advancements. Due to the increasing focus on personalized healthcare and the devastating effect of cardiovascular diseases, there has been rising interest in the field of patient-specific image-based cardiovascular *in silico* modelling. Such models have assisted researchers in gaining a deeper and more complete understanding of cardiovascular diseases and are beginning to assist clinicians in determining personalized and optimal treatments. Moving forward, one of the major challenges in cardiovascular modelling, covering not only fluid dynamics but also electrophysiological modelling for long term patient specific predictions. As the technology develops, researchers and physicians should envision the ultimate objective of a personalized cardiovascular model that incorporates personalized genomics, cellular behavior, tissue structure integrated with cardiovascular mechanics and fluid dynamics. We hope that our readers find the work published here both informative and stimulating in the endeavor to progress the therapy of cardiovascular disease.

